# Annual and geographical variations in the specific composition of jassids and their damage on cotton in Ivory Coast

**DOI:** 10.1038/s41598-024-52127-y

**Published:** 2024-01-24

**Authors:** Houphouet Kouadio, Malanno Kouakou, Kouadio Kra Norbert Bini, Kouakou Jean Innocent Koffi, Christian Landry Ossey, Pitou Woklin Euloge Kone, Abouo Béatrice Adepo-Gourene, Ochou Germain Ochou

**Affiliations:** 1 Unité de Formation et de Recherche des Sciences de la Nature, Laboratoire de Génomique Fonctionnelle et d’Amélioration Génétique (LaGeFAGe), Université Nangui Abrogoua, 02 BP 801, Abidjan, 02 Côte d’Ivoire; 2 Station de Recherche sur le Coton, Laboratoire Entomologie, Centre National de Recherche Agronomique, 01 BP 633, Bouaké, 01 Côte d’Ivoire; 3 Laboratoire Central Sols Eaux et Plantes, Centre National de Recherche Agronomique, 01 BP 633, Bouaké, 01 Côte d’Ivoire; 4 Station de Recherche sur les Cultures Vivrières, Programmes Cultures Maraichères et Protéagineuses, Centre National de Recherche Agronomique, 01 BP 633, Bouaké, 01 Côte d’Ivoire; 5 UFR Ingénierie Agronomique, Forestière et Environnementale, Université de Man, BP 20, Man, Côte d’Ivoire

**Keywords:** Entomology, Biodiversity

## Abstract

In recent years, jassids have become a real problem for cotton growing in Ivory Coast. It is important to investigate the causes of this problem. The aim of this study was to highlight the diversity of jassid species and their impact on cotton growing in Ivory Coast. The collections carried out in 2021 identified three species. *Jacobiasca lybica* (Bergevin & Zanon, 1922) is the most abundant, with proportions ranging from 73.3 to 93.3% depending on the site. The other two species are *Empoasca papayae* (Oman, 1937) (8.3%) and *Empoasca facialis* (Jacobi, 1912) (5%). In 2022, collections revealed the invasion of a new species, *Amrasca biguttula* (Shiraki, 1913), which became dominant with proportions of 90 to 100% depending on the site. Two other species, *Jacobiasca lybica (*Bergevin & Zanon, 1922) (2.7%) and *Empoasca* facialis (Jacobi, 1912) (1.3%), cohabit with *Amrasca biguttula* (Shiraki, 1913). Thus, while the damage noted in 2021 was attributable to *Jacobiasca lybica* (Bergevin & Zanon, 1922*)*, that observed in 2022 is essentially due to *Amrasca biguttula* (Shiraki, 1913), with incidences exceeding the economic impact threshold. The North-East of the cotton basin was the area most affected by attacks by these two species. The results of the study reveal significant changes in jassid species composition and climatic conditions in the cotton-growing areas of Ivory Coast, from 1 year to the next. This situation also explains the variations in damage levels.

## Introduction

Cotton growing is a major source of income for the people living in the Ivorian cotton basin. It is the main economic component and engine of development for this part of Ivory Coast. It plays an important role in the socio-economic development of the populations living there^1^. However, it is subject to numerous pests, among which jassids are playing an increasingly important role. Jassids are sucking, biting insects of the Cicadellidae family. Cicadellidae belong to the order Hemiptera. This order contains a wide variety of species. These insects used to be relegated to the status of minor pests, but for over a decade now they have been a real concern for cotton crops^1,2^.

Indeed, before the 2000s, jassids were considered as emergence pests. Pullulations of these insects were generally observed during the first 40 to 50 days of cultivation. Populations dropped considerably with the start of insecticide treatments on the 45th day after emergence or as soon as the rains began^1^. However, since 2012 these leafhoppers have become emerging pests. Infestation and damage levels remain constantly high on cotton plants throughout the growing season, despite the monitoring and compliance with the phytosanitary protection program applied by growers^1^. These attacks are much more pronounced in the northeastern part of the cotton production zone^2^. These findings have prompted research into crop protection programs adapted to these areas, where sucking biting insects are predominant^3^. It should be emphasized that the protection strategy in force in Ivory Coast is based on a calendar program of six (6) insecticide treatments, every 14 days, from the 45th to the 115th day after cotton emergence. Before starting insecticide treatments, beneficial insects are used to control cotton pests. Thus, starting spraying on the 45th day after cotton emergence enables beneficial insects to naturally regulate cotton pests. One restriction imposed by this strategy is the ban on the use of pyrethroid-based products. This reduces the resistance observed in *Helicoverpa armigera* Hübner to this chemical family^4^. Pyrethroids are banned at the start of the cropping season until August 10 in northern cotton-growing areas (above the 9th parallel) and until August 20 in southern cotton-growing areas (below the 9th parallel).

Despite these actions, during the 2022–2023 crop year, more severe attacks than in previous years were observed throughout the cotton production zone in Ivory Coast and in several neighboring countries. This damage was estimated at around 34 billion franc of the African Financial Community (f CFA).

According to^1^ have shown that pockets of drought during the July–August period favor jassid outbreaks. However, other factors such as substitutions or introductions of new species could also explain these constantly observed changes in behavior. The jassid species known in Ivory Coast are *Empoasca facialis* (Jacobi, 1912) and *Orosius cellulosus* (Lindberg, 1927)^5,6^. The first species is characterized by the injection of toxic saliva into the host plant, resulting in leaf discoloration. The second species is known as the vector of a phytoplasma responsible for floral virescence^1,6–8^. However, the main species known to cause the most damage is *Empoasca facialis* (Jacobi, 1912). Today, we don't know whether this species is still the only or the main cause of the damage observed. Could the emergence of new species in the field be the consequence of the invasion of new species?

The general aim of the present work was to contribute to understanding the causes of regular outbreaks of jassids in cotton crops. Specifically, it aimed to identify wasp species and their dynamics in cotton crops.

## Material and methods

### Materials

The study focused on leafhopper specimens collected on cotton (*Gossypium hirsutum* Linné, 1763) in a production environment. The main cotton varieties collected were CI 128, CI 123, Gouassou and Sicama. They were developed by the Centre National de Recherche Agronomique (CNRA). They are hybrid varieties.

The protection program popularized by growers is the six-treatment insecticide window program. This program reduces the use of pyrethroid-based insecticides by positioning alternatives in the first window. The main active ingredients used for crop protection are listed in Table 1.

**Table 1:** Characteristics of the main active ingredients used in the phytosanitary protection of cotton in Côte d'Ivoire.

Active ingredients	Doses of active ingredients (g/ha)	Chemical family
Cypermethrin + abamectin	36 + 14	Pyrethroid/avermectin
Dinotefuran + alphacypermethrin	11.2 + 17.6	Neonicotinoid/pyrethroid
Spinetoram + sulfoxaflor	14 + 14	Spinosyn/sulfloximin
Spiromesifen	144	Tetronic and tetramic acid derivatives
Deltamethrin + chlorpyrifos ethyl	12 + 200	Pyrethroid/organophosphate
Emamectin + acetamiprid	12 + 16	Avermectin/neonicotinoid
Cypermethrin + profenofos	36 + 300	Pyrethroid/organophosphate
Alphacypermethrin + acetamiprid	18 + 8	Pyrethroid/neonicotinoid
Buprofezin	200	Buprofezin
Chlorantraniliprol	20	Diamide

### Study site

**Figure 1:**
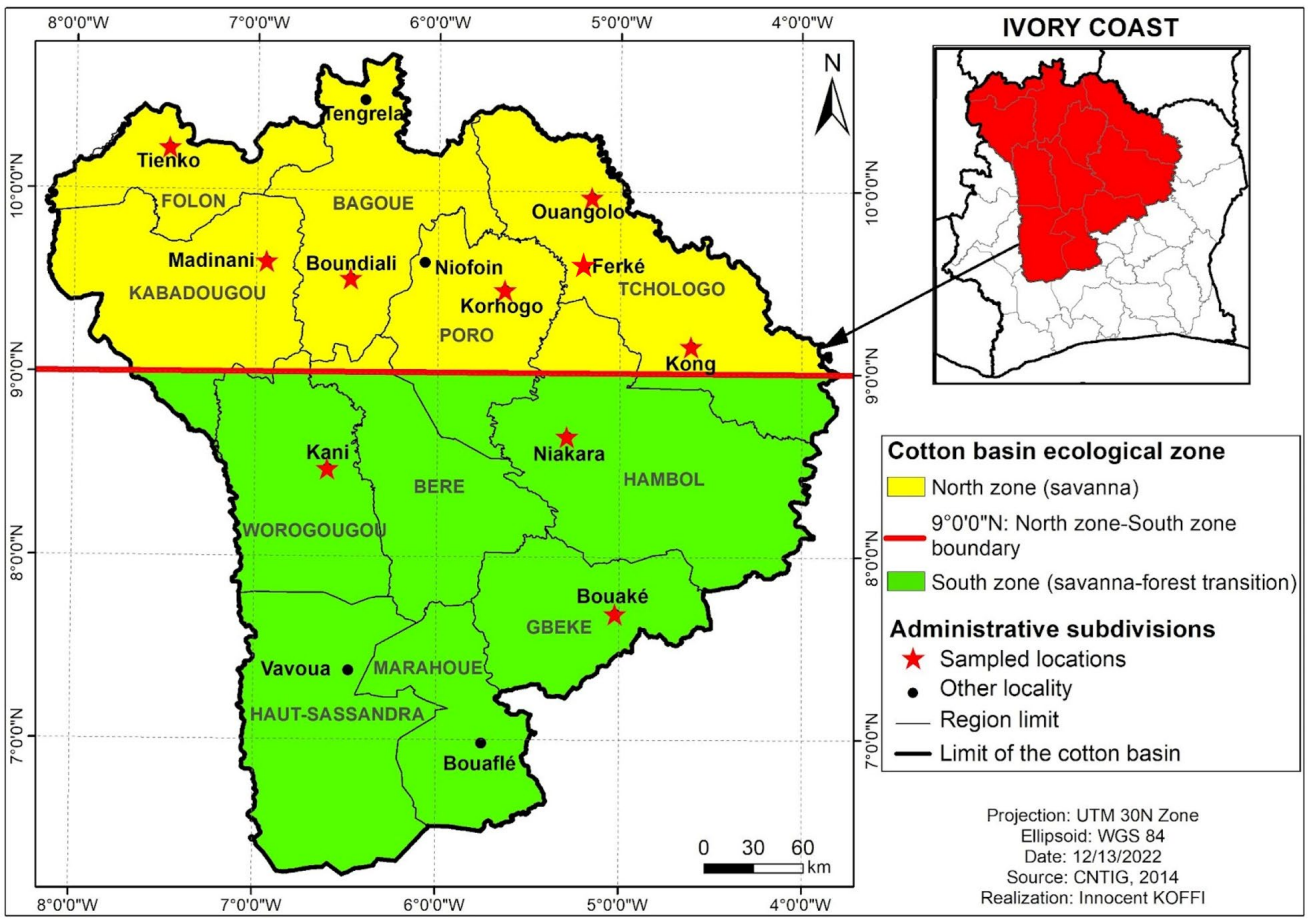
Location of the 10 localities sampled in the Ivorian cotton basin, Arc gis 10.2.2, https://enterprise.arcgis.com/fr/inspire/10.3/get-started/release-notes-10-2-1-for-inspire.htm.

The leafhopper specimens used for species identification were collected in 10 localities in the cotton basin (7°5N to 12°N: 3°W to 8.5°W), namely Tienko, Madinani, Boundiali, Ferké, Kani, Kong, Korhogo, Nambingué (Ouangolo), Niakara, and Bouaké (Fig. 1).

### Sample collections

Specimens were collected for identification during the 2021 and 2022 seasons, from June to November each year. The jassids were caught plant by plant, on a sample of 30 plants taken in groups of five consecutive plants per row, using the sequential "diagonal" method^9^. Adult jassids were collected using a mouth aspirator early in the morning from 7:30 a.m. to 1:00 p.m., then preserved on naphthalene, over a 0.25 ha area marked out in the plot center.

### Identification of individuals

Individuals were identified based on taxonomic keys^10–12^ consolidated by^13^ from various resources. They were dissected and then meticulously observed using a Panthera L Motic microscope at 4× and 10× magnifications respectively, and a Motic SMZ-171-TLED magnifying glass.

### Observation of jassid damage on cotton plants

Data as collected in collaboration with the cotton companies' Research and Development (R&D) departments. Each year, the monitoring system covered around 320 plots in farming areas. These plots were selected in 32 localities, with ten (10) plots per locality. The plots were chosen according to the most representative sowing dates in each locality (Table 2). An area of 0.25 ha was delimited in the Centre of the plot for data collection. Each plot was subjected to the technical practices recommended for cotton production in Ivory Coast (variety, sowing density, fertilization, weeding, etc.).

**Table 2:** Choice of the number of plots according to the sowing decades in the North and South cotton production zones.

Seeding dates	North Region	South Region
D1 (May 20–31)	1	
D2 (June 01–10)	2	1
D3 (June 11–20)	3	3
D4 (June 21–30)	2	3
D5 (July 01–10)	1	2
D6 (July 11–20)	1	1
Total by locality	10	10

On each plot, weekly observations were made from the 30th to the 128th JAL (Jour Après Levée), i.e., for 14 weeks. Observations of plant damage and adult jassids were made plant by plant, on a sample of 30 plants in six groups of five consecutive plants using the diagonal method^9^. A plant is considered attacked when one of its five terminal leaves shows damage or symptoms of jassid attack. Data for 2021 to 2022 were collected using the SySParCotCI data collection application developed by the National Center for Agronomic Research^14^. Data from 2012 to 2020 were collected using the same method, based on the data collection sheets that were used prior to the development of the SySParCotCI data collection application.

### Climatic data collection

Rainfall data were provided by the central soil, water, and plant laboratory of the Centre National de Recherche Agronomique (CNRA). These (in-situ) data are taken every day of the year, from weather stations installed not far from the farmers' plots. As for temperature and relative humidity data, we mobilized them from the *Nasa power* platform (https://power.larc.nasa.gov/data-access-viewer/), which provides access to a range of climatic data available at daily, monthly, and annual time steps. For this study, we focused on data from June to July, during the 2021 and 2022 season.

### Data analysis

After identification, the percentages of individuals belonging to the different species were calculated according to crop year and locality, and an overall average was determined for both crop years simultaneously. The same analyses were carried out according to locality. Geographical and seasonal damage levels were determined from data collected in 2021 and 2022. Average annual damage levels for the last decade (2012 to 2022) were also determined by combining data collected from 2012 to 2022. Average annual damage levels for the last decade (2012 to 2022) have also been determined by combining data collected from 2012 to 2022. Ecological diversity indices were determined using the *Vegan* package for R.^15^, based on data from the 2 years combined. These were the species richness index (S), the Shannon index (H), the dominance index (D), the Pielou equitability index (J) and the Jaccard index (I)^16–18^. The species richness index (S) represents the number of species in a community, regardless of their relative abundance. The Shannon index (H) is a measure of community stability. It varies according to the number of species and the relative proportion of different species. Simpson index (D) measures the probability that two individuals drawn at random from a sample belong to the same species. Pielou equitability index (J) measures the distribution of individuals within species. It is useful for comparing potential dominance between samples. Jaccard index (I) enables a comparison between two sites, as it evaluates similarity by calculating the ratio between species common to both sites and those specific to each survey. Seasonal variation curves were plotted using Excel software from the Windows Office 2016 package. Analyses and graphical representations were carried out using the free Rstudio development environment of the R 4.2.2 software package^19^ using the *questionr* and *ggplot2* packages. The questionr package was used to determine species percentages according to locality and crop year. The *ggplot2* package was used for graphical representations. Spatialization of damage and rainfall levels was carried out using Arc gis 10.2.2 software^20^.

### Ethics

The method of collecting insect specimens was approved by the entomology laboratory of the cotton program of the Centre Nationale de Recherche Agronomique (CNRA). Specimens were collected from non-endangered species. Specimens are kept in the CNRA laboratory. They were collected in cotton growers' fields, in strict compliance with IUCN procedures and regulations.

Experimental research and field studies on plants (either cultivated or wild), including the collection of plant material, complied with relevant institutional, national, and international guidelines and legislation.

### Approval and consent to participate

The authors alone are responsible for the content and writing of this article.

## Results

### Ecological community structure

A total of 600 individuals were identified, including 300 during the 2021 season and 300 during the 2022 season.. At the 10 sites sampled, species richness (S) ranged from three (3) to five (5) species (Table 3). Species diversity determined using the Shannon index (H) ranged from 0.84 (Korhogo) to 1.11 (Boundiali) (Table 3). As the H index is greater than 0, this indicates a high level of species diversity. Piélou's equitability index (J) varied from 0.61 in Korhogo to 0.90 in Bouaké (Table 3). This variation, which differs from the 0 value, shows that the distribution of species in the different localities sampled appears to be identical. As for Jaccard index (I), values varied from 0.50 to 1 (Table 4). Although the I-indices varied from one site to another, values close to 1 showed that the composition of the species found at the different sites was almost identical. The dominance index or simpson index (D) varied from species to species. It was 0.90, 0.90, 0.83, 0.78 and 0.76 respectively for *Amrasca biguttula* (Shiraki, 1913), *Jacobiasca lybica* (Bergevin & Zanon, 1922), *Empoasca papayae* (Oman, 1937), *Cicadellidae sp* and *Empoasca facialis* (Jacobi, 1912). The first two species had the highest probability of occurrence. They reveal their abundance in the samples collected.

**Table 3:** Three ecological diversity indexes (Species richness index, Shannon index, and Pielou equitability index) of leafhoppers collected from 10 localities in the cotton basin in Ivory coast.

Locality	Species richness index (S)	Shannon index (H)	Pielou equitability index (J)
Bouaké	3	0.99	0.90
Boundiali	5	1.11	0.69
Ferké	4	1.04	0.75
Kani	5	1.09	0.68
Kong	5	0.99	0.62
Korhogo	4	0.84	0.61
Madinani	5	1.00	0.62
Nambingué	4	0.93	0.67
Niakara	3	0.77	0.70
Tienko	3	0.86	0.78

**Table 4:** Index of presence (Jaccard index) of leafhoppers collected in 10 localities in the Côte d'Ivoire cotton basin.

	Bouaké	Boundiali	Ferké	Kani	Kong	Korhogo	Madinani	Nambingué	Niakara
Boundiali	0.60								
Ferké	0.75	0.80							
Kani	0.60	1.00	0.80						
Kong	0.60	1.00	0.80	1.00					
Korhogo	0.40	0.80	0.60	0.80	0.80				
Madinani	0.60	1.00	0.80	1.00	1.00	0.80			
Nambingué	0.75	0.80	1.00	0.80	0.80	0.60	0.80		
Niakara	1.00	0.60	0.75	0.60	0.60	0.40	0.60	0.75	
Tienko	0.50	0.60	0.75	0.60	0.60	0.75	0.60	0.75	0.50

### Main leafhopper species identified over the period 2021–2022

The leafhopper species identified on cotton are divided into three genera (Table 5). These are the *Empoasca*, *Amrasca* and *Jacobiasca* genera. The main species encountered are: *Empoasca papayae* (Oman, 1937), *Jacobiasca lybica* (Bergevin & Zanon, 1922), *Empoasca facialis* (Jacobi, 1912) and *Amrasca biguttula* (Shiraki, 1913).

**Table 5:** Relative proportion of the main leafhopper species identified on cotton in Ivory Coast.

Genus	Species	Relative proportion (%)
*Empoasca*	*Empoasca papayae* (Oman, 1937)	4.3
	*Empoasca facialis (*Jacobi, 1912)	3.2
*Jacobiasca*	*Jacobiasca lybica* (Bergevin & Zanon, 1922)	43.5
*Amrasca*	*Amrasca biguttula* (Shiraki, 1913)	48

### Annual variation in overall species composition of leafhopper species

The proportion of species identified varied from one season to the next (Table 6). In 2021, the majority of species identified were *Jacobiasca lybica* (Bergevin & Zanon, 1922) with an overall proportion of 84.7%. It was followed by *Empoasca papayae* (Oman, 1937) and *Empoasca facialis* (Jacobi, 1912). Their average proportions were 8.7% and 5% respectively. In 2022, *Amrasca biguttula* (Shiraki, 1913) was the most abundant species, with an overall proportion of 96%. It was followed by Jacobiasca lybica (Bergevin & Zanon, 1922) and *Empoasca facialis* (Jacobi, 1912). Their overall proportions were 2.7% and 1.3% respectively. The species *Empoasca papayae* (Oman, 1937) was not found in the samples collected in 2022. The same applies to *Amrasca biguttula* (Shiraki, 1913), which was not observed during the 2021 season.

**Table 6:** Annual variation in the overall specific composition of leafhoppers during the 2021 and 2022 cropping seasons in Ivory Coast.

Genus	Species	Overall proportion (%)	
		2021	2022
Empoasca	*Empoasca papayae* (Oman, 1937)	8.7	0
	*Empoasca facialis (*Jacobi, 1912)	5	1.3
Jacobiasca	*Jacobiasca lybica* (Bergevin & Zanon, 1922)	84.3	2.7
Amrasca	*Amrasca biguttula* (Shiraki, 1913)	0	96
	*Cicadellidae* sp.	2	0

### Geographic variation in overall species composition

The proportions of different species varied among localities (Table 7). Two species were found to be in the majority: *Amrasca biguttula* (Shiraki, 1913) and *Jacobiasca lybica* (Bergevin & Zanon, 1922). Their proportions varied respectively from 45 to 50% and from 40 to 50% according to the localities. However, *Amrasca biguttula* (Shiraki, 1913) was the most abundant.

**Table 7:** Geographical variation in the proportions of leafhopper species identified in the 10 localities sampled in Ivory Coast.

Locality	*Amrasca biguttula* (Shiraki, 1913*)*	*Jacobiasca lybica* (Bergevin & Zanon, 1922)	*Empoasca facialis* (Jacobi, 1912)	*Empoasca papayae* (Oman, 1937)	*Cicadellidae *sp.
Bouaké	45	41.7	13.3	0	0
Boundiali	46.7	40	3.3	8.3	1.7
Ferké	46.7	40	1.7	11.7	0
Kani	48.3	40	3.3	5	3.3
Kong	50	41.7	1.7	5	1.7
Korhogo	48.3	48.3	0	1.7	1.7
Madinani	46.7	45	5	1.7	1.7
Nambingué	50	43.3	1.7	5	0
Niakara	48.3	50	1.7	0	0
Tienko	50	45	0	5	0

### Dynamics of geographic variation in species composition between 2021 and 2022

From 2021 to 2022, leafhopper species encountered on cotton varied by locality and crop year (Table 8). However, in both crop years, three main species were identified. In 2021, *Jacobiasca lybica* (Bergevin & Zanon, 1922) was the most dominant species with a proportion ranging from 73.3 to 96.7%. However, in 2022 it was reduced to a proportion ranging from 0 to 6.7% depending on the locality, making it the second most dominant species during this season. *Empoasca papayae* (Oman, 1937) increased from 0 to 23.3% in 2021, then decreased to 0% in 2022, depending on locality, making it an extinct species in this season. *Empoasca facialis* (Jacobi, 1912) was the minority species in 2021, with a proportion ranging from 0 to 16.7% and decreased in 2022 to a proportion ranging from 0 to 10% depending on the locality. It remained the minority species. However, this survey identified it in only two localities, including Bouaké and Boundiali. *Amrasca biguttula* (Shiraki, 1913) went from being absent from the samples collected in 2021 (0%) to the most abundant species in 2022, with a proportion ranging from 90 to 100%, depending on the locality.

**Table 8:** Geographical dynamics of leafhopper species proportions during the 2021 and 2022 cropping seasons in 10 localities in Ivory Coast.

Locality	2021				2022		
	*Jacobiasca lybica* (Bergevin & Zanon, 1922) (%)	*Empoasca papayae* (Oman, 1937) (%)	*Empoasca facialis* (Jacobi, 1912) (%)	*Cicadellidae sp* (%)	*Amrasca biguttula* (Shiraki, 1913) (%)	*Jacobiasca lybica* Bergevin & Zanon, 1922) (%)	*Empoasca facialis* (Jacobi, 1912) (%)
Bouaké	83.3	0	16.7	0	90	0	10
Boundiali	76.7	16.7	3.3	3.3	93.3	3.3	3.3
Ferké	73.3	23.3	3.3	0	93.3	6.7	0
Kani	76.7	10	6.7	6.7	96.7	3.3	0
Kong	83.3	10	3.3	3.3	100	0	0
Korhogo	93.3	3.3	0	3.3	96.7	3.3	0
Madinani	83.3	3.3	10	3.3	93.3	6.7	0
Nambingué	86.7	10	3.3	0	100	0	0
Niakara	96.7	0	3.3	0	96.7	3.3	0
Tienko	90	10	0	0	100	0	0

### Geographic variation in infestation levels

**Figure 2:**
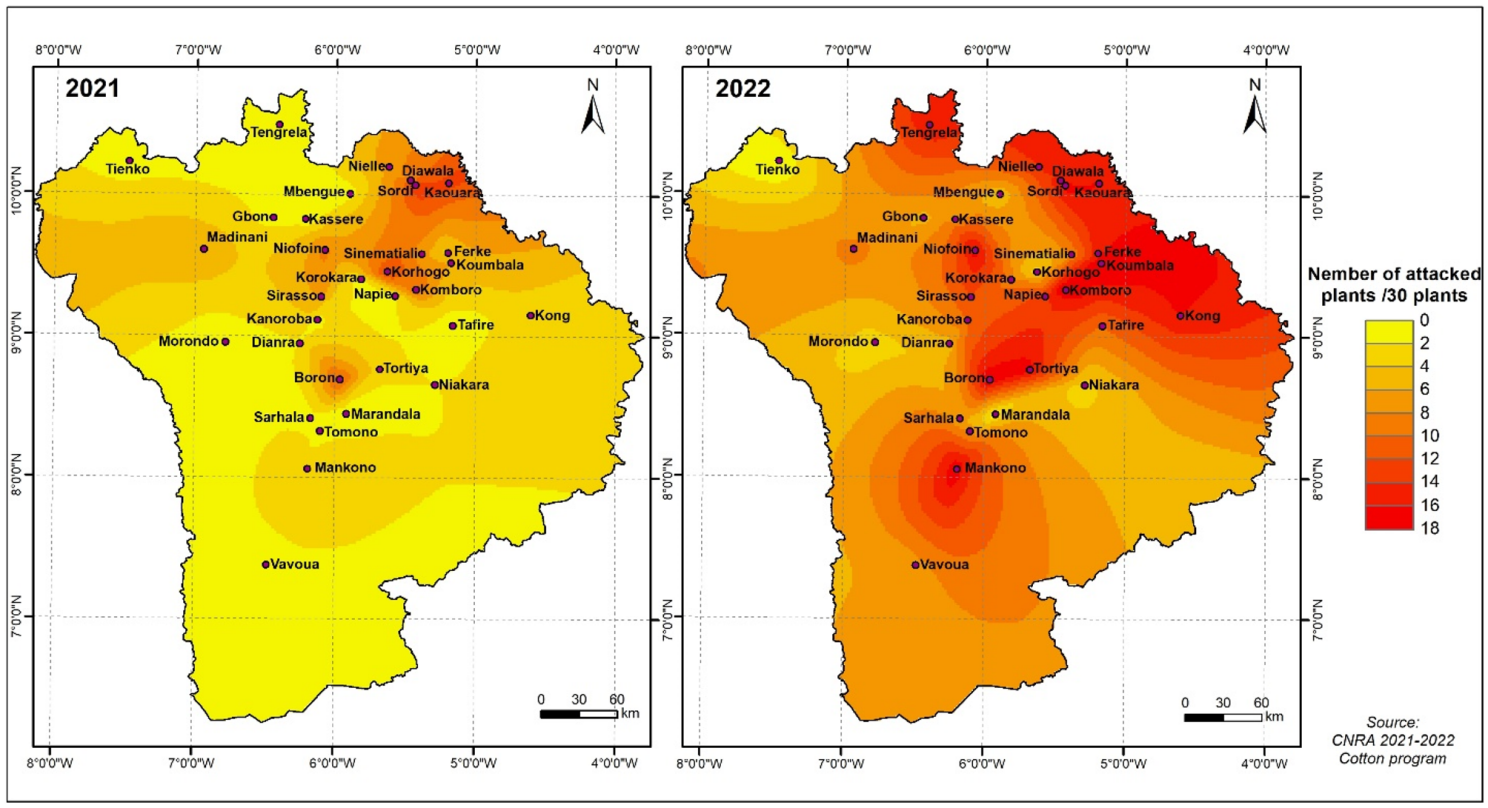
Geographical variation in leafhopper damage in 32 localities in the cotton basin, Arc gis 10.2.2, https://enterprise.arcgis.com/fr/inspire/10.3/get-started/release-notes-10-2-1-for-inspire.htm.

The levels of leafhopper damage varied among localities in the two crop years (Fig. 2). In the 2021 season, attacks were significant in the northern part of the cotton basin. However, they were particularly pronounced in the northeast of the cotton basin. During the 2022 season, attacks were widespread throughout the cotton basin. However, the highest infestations were observed in the northern part of the cotton basin, with higher levels in the northeastern part.

### Seasonal and annual variation in infestation levels

**Figure 3:**
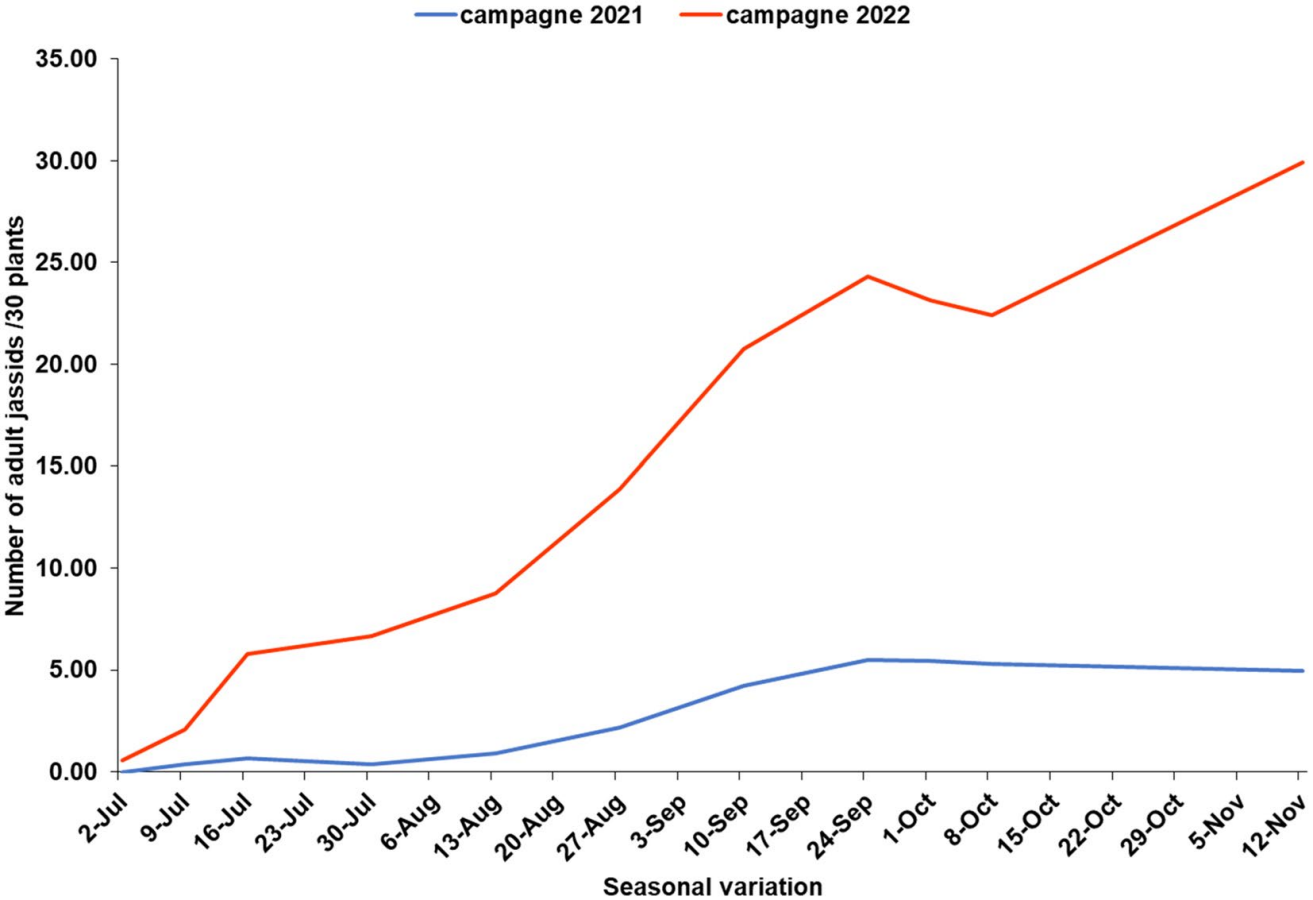
Seasonal variation in adult jassid infestation levels observed during the 2021 and 2022 crop year.

Seasonal incidence of leafhoppers varied in both crop years (Fig. 3). In both years, attacks increased throughout the cropping season, but with higher levels in 2022 (more than 30 adult jassids/30 plants).

**Figure 4:**
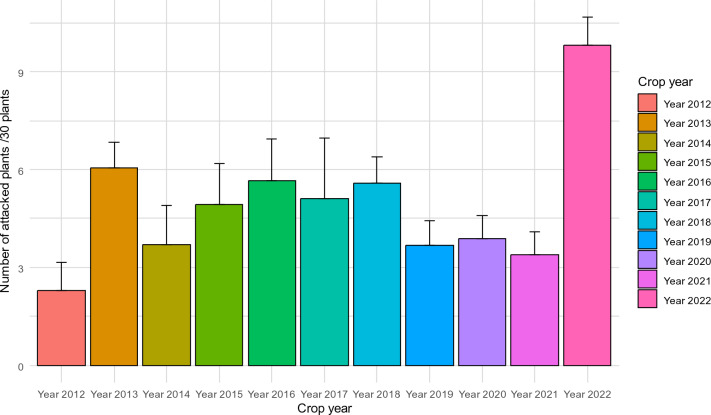
Annual variation in levels of damage caused by jassid attacks observed over the last 10 years in the Ivorian cotton basin.

Leafhopper attacks varied by year from 2012 to 2022 (Fig. 4). From 2012 to 2021, average attack levels ranged from 2.3 to 6.04 plants attacked per 30 plants observed. These levels were relatively low compared to those observed in 2022. Indeed, the average level of attacks was more than nine attacked plants per 30 plants observed in 2022. This level had never been reached in the previous 10 years (Fig. 4).

### Geographical variation in rainfall

**Figure 5:**
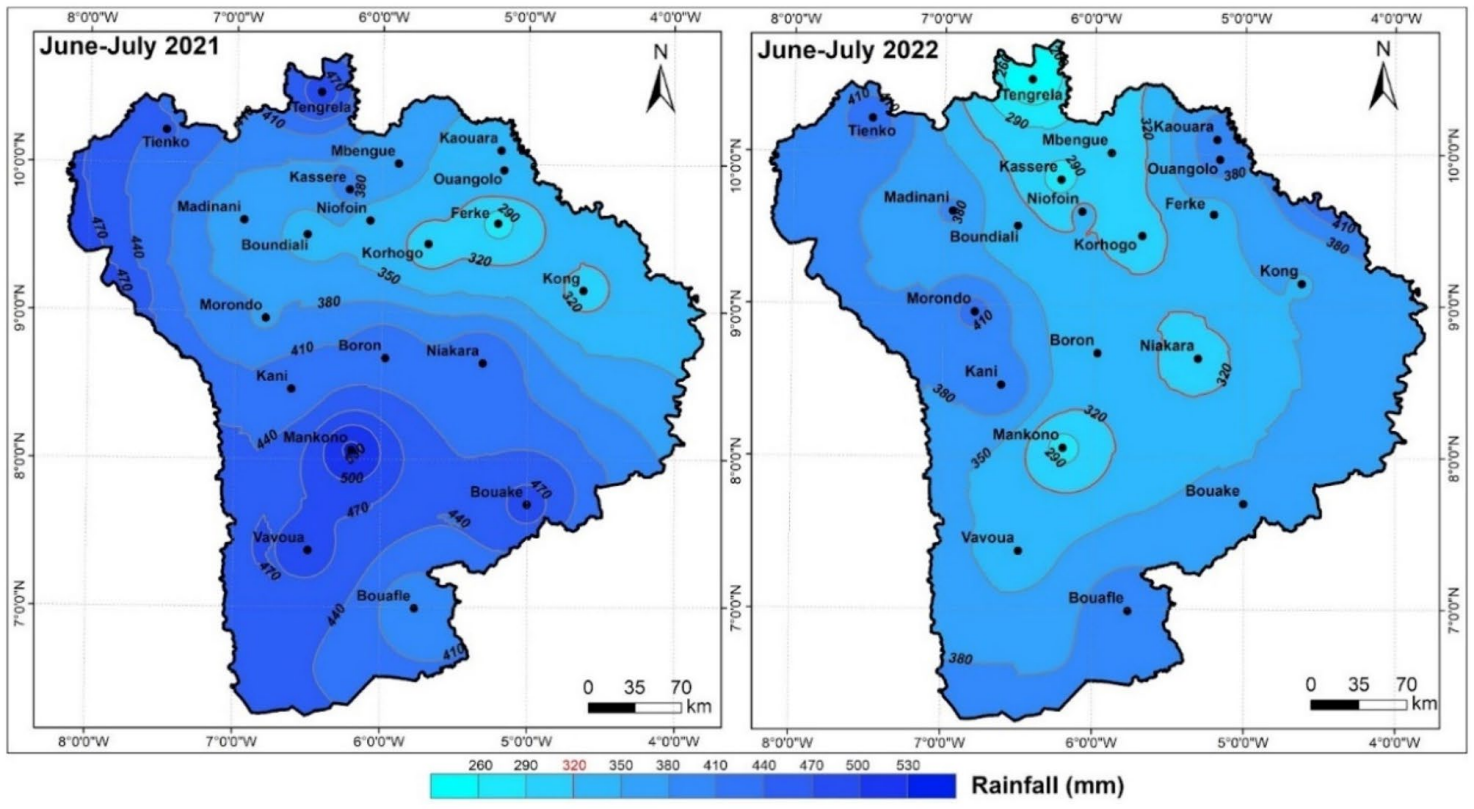
Geographical variation in rainfall from June to July during the 2021 and 2022 crop years, Arc gis 10.2.2, https://enterprise.arcgis.com/fr/inspire/10.3/get-started/release-notes-10-2-1-for-inspire.htm.

Cumulative rainfall from June to July varied downwards from 2021 to 2022 (Fig. 5). During the 2021 season, cumulative rainfall varied between 290 and 530 mm, following the North-East/South-West gradient. In 2022, however, the cumulative rainfall varied between 260 and 410 mm, a drop ranging from 30 to 120 mm. In addition to the drop in rainfall, a geographical peculiarity can be observed in that the lowest values were recorded in the Tangrela-Mankono-Kong triangle, covering the North-Central-East zone, with an extreme of less than 260 mm recorded in Tengrela.

### Geographical variation in relative humidity

**Figure 6:**
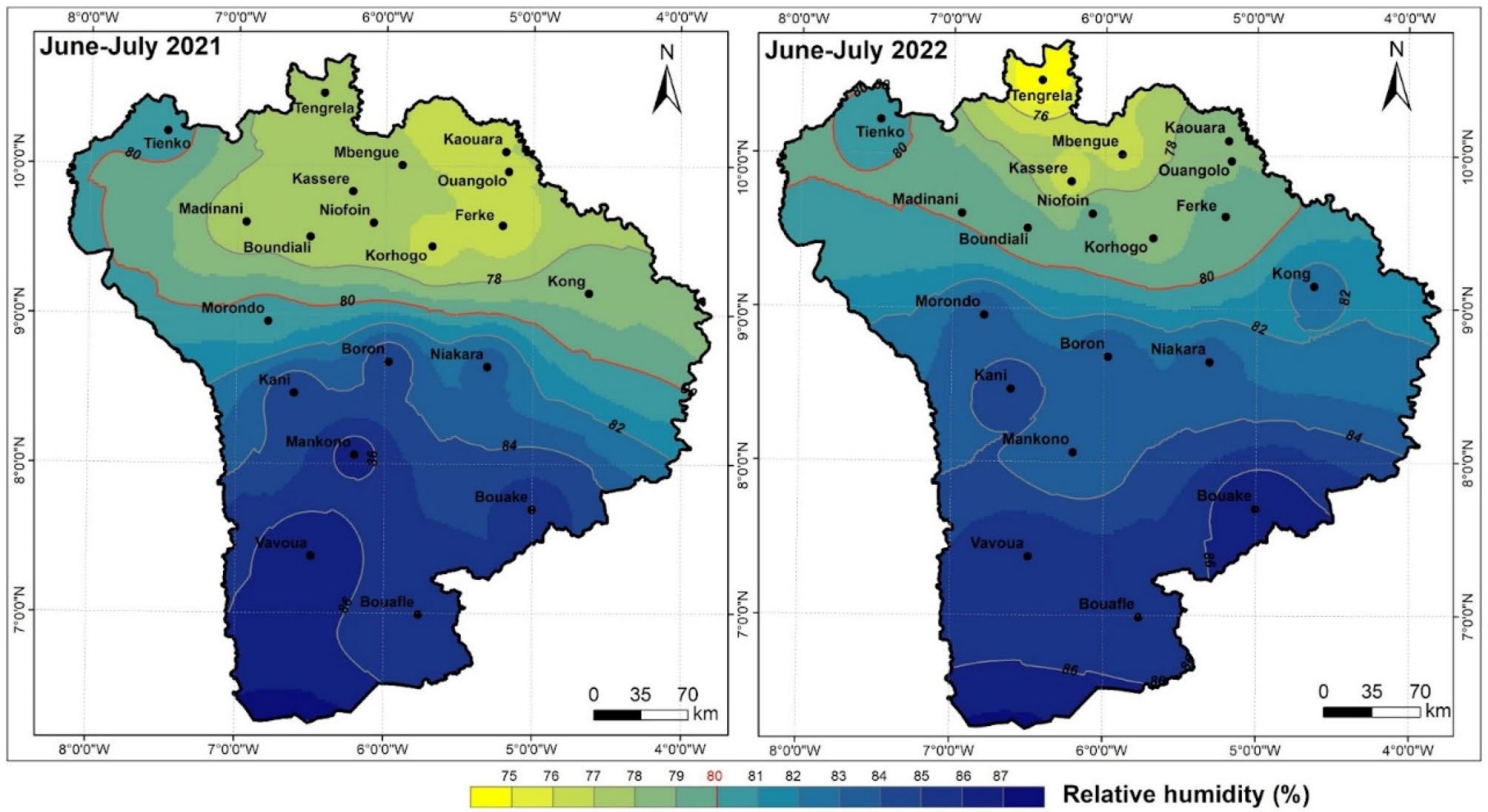
Geographical variation in relative humidity from June to July during the 2021 and 2022 crop years, Arc gis 10.2.2, https://enterprise.arcgis.com/fr/inspire/10.3/get-started/release-notes-10-2-1-for-inspire.htm.

In the cotton basin, average relative humidity from June to July generally varied downwards from south to north, from 2021 to 2022 (Fig. 6). Over the two crop years, it varied between 77–87% (2021) and 75–87% (2022), a decrease of 2% from Tengrela (North) to Bouaflé (South). However, beyond the 9th parallel, the 2021 season recorded average relative humidity levels varying between 77 and 80%, following a North-East/South-West gradient, while in 2022, the variation was between 74% (Tengrela) and 80%, with the exception of the Kong zone, which recorded an average humidity level of 82%.

### Geographical temperature variation

**Figure 7:**
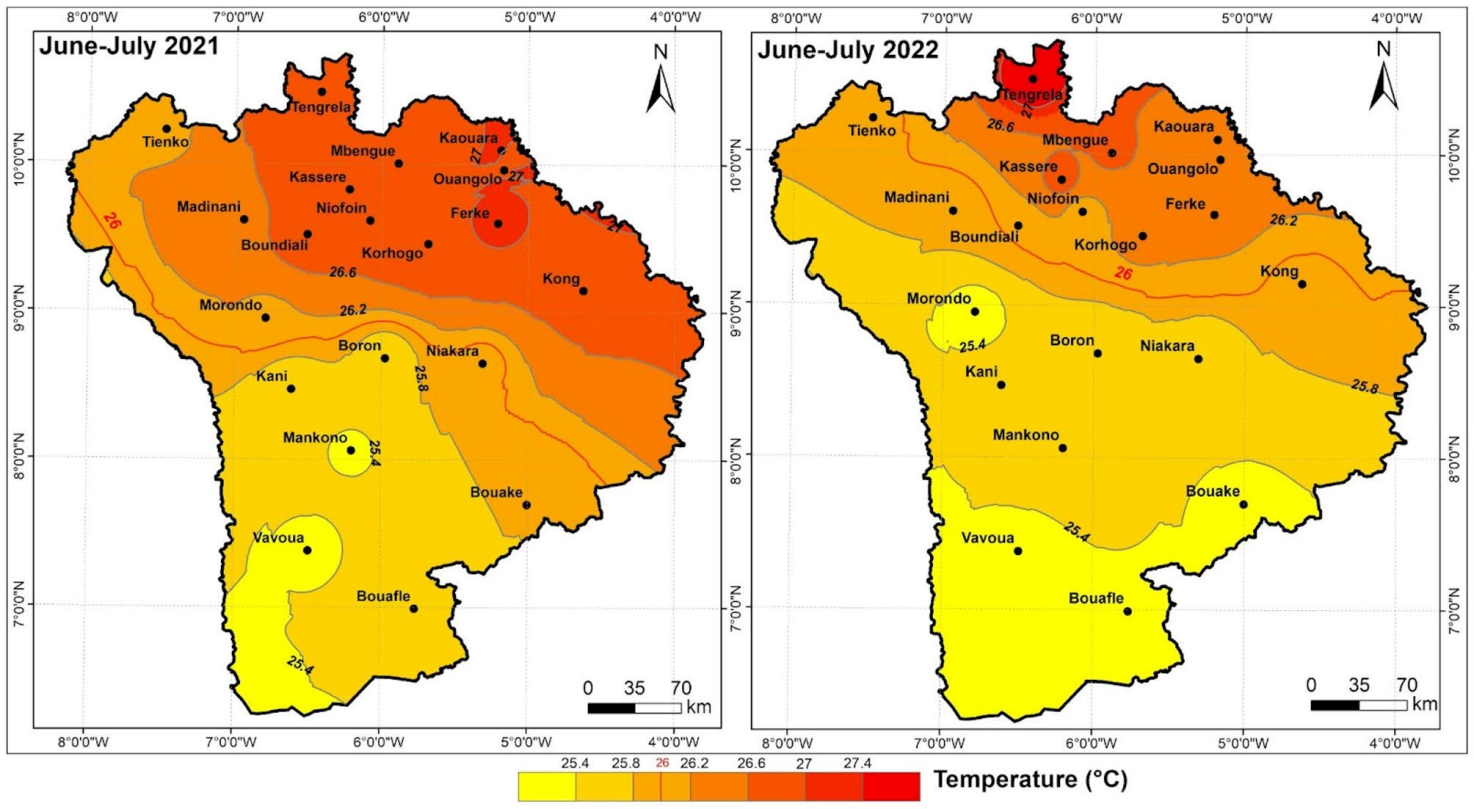
Geographical variation in temperature from June to July during the 2021 and 2022 crop years, Arc gis 10.2.2, https://enterprise.arcgis.com/fr/inspire/10.3/get-started/release-notes-10-2-1-for-inspire.htm.

From 2021 to 2022, the average temperature from June to July fell from 25 to 27 °C, following a north-east/south-west gradient (Fig. 7). Beyond the 9th parallel, there was a reduction in the high-temperature zone from 2021 to 2022. In 2021, the zone is dominated by an average temperature of over 26.6 °C, compared with 26.2 °C in 2022.

## Discussion

Indices of ecological diversity and analysis of annual and geographical variation have revealed a diversity of species that varies according to crop year and locality. In fact, during the 2021 crop year, identifications revealed three main jassid species. Two of these species had not been identified in the first pest surveys carried out in Ivory Coast^5,6^. However, preliminary identifications carried out in 2019 by the entomology laboratory of the National Agricultural Research Centre's cotton program highlighted these species. These were *Jacobiasca lybica* (Bergevin & Zanon, 1922) and *Empoasca papayae* (Oman, 1937). However, the first species proved to be the most dominant and invasive, occupying a larger proportion than the species native to Ivory Coast (*Empoasca facialis* (Jacobi, 1912)). It accounted for over 80% of all jassid species. The attacks observed during the 2020–2021 crop year could therefore be attributed mainly to *Jacobiasca lybica* (Bergevin & Zanon, 1922). These attacks manifested themselves as leaf discoloration, revealing yellow patches on the leaves.

The 2022 crop year also saw the appearance of a new species in the cotton pest complex. It was identified as *Amrasca biguttula* (Shiraki, 1913), a jassid of Indian cotton^21^. It accounted for 90% to 100% of jassid species, depending on locality. This more invasive and virulent species, which until now had not been reported, would be at the root of the high levels of infestation observed during the 2022 crop year. Indeed, in 2022, damage was more remarkable and widespread throughout the cotton basin, with levels higher than in the 2021 crop year. Over the course of the growing season, infestation levels were constantly above the economic damage threshold (10 plants attacked out of 30 observed). At certain periods, levels reached 100% of plants. This species had a very significant negative impact on growers' income. Losses were estimated at over 34 billion franc of the African Financial Community (f CFA). Damage manifested itself in the total drying-up of cotton plant leaves (the *hopperburn* phenomenon).

The two main species identified in this study were also identified in 2022 by^22^ on okra and eggplant in similar proportions. These species therefore parasitize several host plants, notably eggplant and okra^22,23^. *Amrasca biguttula* (Shiraki, 1913) was first reported in Africa in Ghana on okra as a minor pest^24^. Climatic conditions could favour its expansion in the sub-region, as there are no geographical boundaries for these pests.

Damage from jassids also varied from one locality to another. However, damage was most pronounced in the north-east of the cotton-growing basin. The greatest damage was recorded in this part of the cotton-growing zone. They also varied from 2012 to 2022. However, the greatest damage was observed during the 2022 season. This could be due to the diversity observed, as well as lower rainfall and higher temperatures. Indeed^25,26^, have shown that low rainfall combined with high temperatures and low relative humidity contribute to the healthy development of jassids. Climatic factors therefore play an essential role in the composition and outbreak of jassid populations and in the manifestation of damage^1,26^. These conditions could create a favorable environment for the development of new species not previously present in the cotton basin pest complex. Indeed, the rise and fall in temperatures in July play a role in jassid outbreaks.

The appearance of these species, new to the Ivorian parasite facies, would explain the emergence of jassids in recent years. It would also explain the inadequacy of control programs observed in the field.

## Conclusion

The present study revealed a variation in the diversity of jassid species. Three (3) to five (5) jassid species were identified in the cotton basin. This abundance follows a geographical and annual dynamic according to the crop year, resulting in the appearance of more invasive and virulent species with an adaptability that is favoured by climatic factors.

The most abundant species observed during this study period were *Jacobiasca lybi*ca (Bergevin & Zanon, 1922), identified during the 2021 campaign, and *Amrasca biguttula* (Shiraki, 1913), identified during the 2022 campaign. Both appear to be invasive. However, *Amrasca biguttula* (Shiraki, 1913) appears to be the most invasive and virulent. Its appearance has led to the virtual extinction of certain species in certain localities. It has thus disrupted the pest complex in cotton-growing areas, with damage levels exceeding the economic impact threshold throughout the cotton-growing basin. It has had a significant impact on cotton growing.

It is planned to continue identifying species using molecular tools in order to extend knowledge of any species that could not be identified using conventional taxonomy. Bio-ecological studies on the life cycle of *Amrasca big*uttula (Shiraki, 1913), its natural enemies and the susceptibility of cotton varieties and vegetable crops to jassids are also envisaged, in order to contribute to the implementation of an ecological control strategy.

## Data Availability

Data and materials are available on request from the author Houphouet KOUADIO at the following addresses: houphouet.kouadio@gmail.com.
